# Personality and Coming to Terms with Cancer: A Cross-Sectional Study of Five-Factor Traits and Illness Acceptance in Oncology Outpatients

**DOI:** 10.3390/jcm15187008

**Published:** 2026-09-10

**Authors:** Robert Jan Łuczyk, Dorota Weber, Agata Wiśnios, Marta Łuczyk, Kamil Sikora, Anna Charuta

**Affiliations:** 1 Institute of Health Sciences, Faculty of Medical and Health Sciences, University of Siedlce, 08-110 Siedlce, Poland; anna.charuta@uws.edu.pl; 2 Faculty of Health Sciences, Lublin University of Technology, 20-209 Lublin, Poland; dorota.weber@wsei.pl; 3 Faculty of Health Sciences, WSEI University in Lublin, Projektowa 4, 20-209 Lublin, Poland; 4 Department of Internal Medicine and Internal Nursing, Chair of Preventive Nursing, Faculty of Health Sciences, Medical University of Lublin, Ul. Chodźki 7, 20-093 Lublin, Polandkamil.sikora@umlub.edu.pl (K.S.); 5 Long-Term Care Nursing Department, Chair of Preventive Nursing, Faculty of Health Sciences, Medical University of Lublin, Ul. Chodźki 7, 20-093 Lublin, Poland; marta.luczyk@umlub.edu.pl

**Keywords:** cancer, oncology, malignant tumor, personality, Five-Factor Model, NEO-FFI, illness acceptance, Acceptance of Illness Scale, social support, psycho-oncology

## Abstract

**Background/Objectives:** A cancer diagnosis forces a rapid, often unwelcome reorganization of a patient’s sense of self, routines, and future plans, and patients vary considerably in how far they progress toward accepting that new reality. Personality is a candidate explanation for this variability, and although the Five-Factor Model has been linked to cancer-related distress and coping in several large cohorts, its relationship to illness acceptance specifically, alongside disease duration, treatment self-assessment, and perceived social support, has not been jointly examined in a single, diagnostically broad oncology sample. **Methods:** We surveyed 114 outpatients with a confirmed malignant tumor diagnosis, combining a study-specific questionnaire with the NEO-Five-Factor Inventory (NEO-FFI) and the Acceptance of Illness Scale (AIS). Because several study variables were non-normally distributed and the study-specific measures were ordinal, associations were examined using Spearman’s correlation, the Kruskal–Wallis H test, and the Mann–Whitney U test. **Results:** Illness acceptance was moderate on average (mean [M] = 26.55, standard deviation [SD] = 7.34); 22 patients (19.30%) were classified as having low acceptance, 76 (66.67%) as average acceptance, and 16 (14.04%) as high acceptance. All five personality domains correlated with acceptance, led by a strong negative association with neuroticism (ρ = −0.632, *p* < 0.001) and moderate positive associations with extraversion, agreeableness, conscientiousness, and openness to experience (all *p* < 0.001). Longer disease duration and a more favorable self-rated treatment outcome were both associated with higher acceptance, and patients who felt supported by close relatives scored higher than those who found support difficult to gauge. **Conclusions:** Personality traits, particularly neuroticism, were associated with illness acceptance. Brief psychosocial assessment may help identify patients reporting greater difficulties in psychological adjustment to cancer.

## 1. Introduction

Cancer remains one of the leading causes of death worldwide, with an estimated 20 million new diagnoses and close to 10 million deaths occurring globally each year [1]. Survival statistics, however, tell only part of the story. For most patients, a cancer diagnosis is also a psychological rupture: established routines, occupational plans, and assumptions about the future are all thrown into question in the space of a single clinic visit, and the months that follow demand not only medical treatment but a slow, often difficult process of psychological adjustment.

That adjustment process has occupied psycho-oncology researchers for decades. An early and influential line of work proposed that a specific personality style—labeled Type C and characterized by emotional suppression, excessive compliance, and difficulty asserting one’s own needs—shaped how patients responded to cancer and its treatment [2]. Related instruments developed around the same period, such as the Mental Adjustment to Cancer scale, operationalized patients’ cognitive and behavioral responses to diagnosis along dimensions including fighting spirit, helplessness, and anxious preoccupation [3], cementing the idea that psychological response to cancer is measurable, variable across patients, and clinically consequential.

Contemporary personality psychology has largely moved from typologies like Type C toward the dimensional Five-Factor Model, which describes stable individual differences along five broad domains: neuroticism, extraversion, openness to experience, agreeableness, and conscientiousness [4,5]. Unlike mood, which shifts with treatment cycles and clinical news, these traits are present well before diagnosis and provide a relatively fixed lens through which each subsequent medical encounter is filtered. Across the general population and chronic-disease samples alike, this five-domain structure has repeatedly predicted long-term health, well-being, and even survival [6], with neuroticism singled out as a trait of particular public-health relevance because of its broad associations with both physical and psychiatric outcomes [7]. Meta-analytic work spanning thousands of participants has linked the Five-Factor domains to symptoms of clinical anxiety, depressive, and substance-use disorders [8,9], while conscientiousness in particular has been tied to the health behaviors that most directly drive mortality risk [10].

Within oncology specifically, the largest study to date—a latent-profile analysis of NEO-FFI scores in 1248 patients undergoing chemotherapy for breast, gastrointestinal, gynecological, or lung cancer—identified three personality-based subgroups. A “Distressed” class, marked by the highest neuroticism and the lowest extraversion, agreeableness, and conscientiousness, reported significantly more depression, anxiety, and cancer-related symptoms than an intermediate “Normative” class or a “Resilient” class defined by the opposite personality profile [11]. Follow-up analyses of the same cohort linked these personality profiles to distinct coping repertoires—the Distressed class relied more heavily on denial, self-blame, and behavioral disengagement—and to divergent stress and resilience trajectories over the course of treatment [12,13]. Smaller, more targeted studies echo this pattern: higher neuroticism has been linked to greater demoralization and poorer quality of life among end-of-life cancer patients [14]; to elevated Type-D-related distress in breast cancer [15]; and, in a case–control design, to a higher likelihood of breast cancer diagnosis itself, alongside high openness to experience [16]. Smoking, a major risk factor for several of the cancer sites represented in oncology clinics, has its own personality signature that partly overlaps with this picture: habitual smokers tend to score higher on neuroticism and lower on conscientiousness, extraversion, and openness than people who have never smoked [17,18].

Almost all of this evidence, however, concerns mood, coping style, distress, or general quality of life rather than illness acceptance specifically—the extent to which a patient has stopped resisting the diagnosis and begun to reorganize daily life around it. How a patient makes cognitive and emotional sense of a new diagnosis in the first place has long been recognized as a determinant of downstream adjustment [19], and illness acceptance, measured with instruments such as the Acceptance of Illness Scale, is itself an established predictor of adherence and psychological well-being across chronic conditions [20]. Whether the Five-Factor domains relate to this specific outcome, and how strongly relative they are to more obviously clinical variables such as disease duration, treatment outcome, and perceived social support, remains an open, practically relevant question.

This study addresses that question directly in a diagnostically heterogeneous sample of oncology outpatients: we tested the association between each of the five NEO-FFI personality domains and illness acceptance, alongside disease duration, self-assessed treatment outcome, and perceived support from close relatives.

## 2. Materials and Methods

### 2.1. Setting and Participants

Data collection took place between March and May 2026 at the Department of Diagnostic Imaging of the Oncology Centre of the Lublin Region [Centrum Onkologii Ziemi Lubelskiej im. św. Jana z Dukli], Lublin, Poland. The inclusion criteria were: (1) age ≥ 18 years; (2) outpatient status at the study site; (3) a confirmed diagnosis of a malignant tumor; (4) ability to understand the Polish-language questionnaires; and (5) provision of informed consent. Patients were excluded if they did not meet any of these criteria or returned questionnaires with incomplete NEO-FFI or AIS data. No additional clinical exclusion criteria were applied. Participants were recruited using convenience sampling. Eligible participants were adult oncology outpatients with a confirmed diagnosis of a malignant tumor who were able to understand the Polish-language questionnaires and provide informed consent. During the study period, 150 patients were invited to participate. Of these, 132 agreed to participate and returned questionnaires (participation rate: 88.0%); 18 questionnaires were excluded because of incomplete data, leaving 114 participants in the final analysis (complete-response rate: 76.0%). No additional exclusion criteria were applied. All 114 respondents had a confirmed diagnosis of a malignant tumor. Every patient approached was informed in advance that participation was voluntary and anonymous and that responses could not be traced back to individual patients; the questionnaire packet opened with a short explanation of the study’s purpose and instructions for completing it correctly. Participants generally completed the paper-and-pencil questionnaires independently. When assistance was required because of visual or physical limitations, a member of the research team read the questions verbatim and recorded the responses selected by the participant, without interpreting the items or suggesting answers.

### 2.2. Instruments

The survey packet combined a study-specific questionnaire with two validated psychometric scales. The original questionnaire comprised 10 items, mostly closed and single-choice, covering sociodemographic characteristics, tumor site (free-text or selected from a list), self-assessed treatment outcome, perceived support from close relatives, and beliefs about whether personality shapes coping with illness; one open-ended item recorded disease duration in years.

Personality was assessed using the Polish-language adaptation of the NEO-Five-Factor Inventory (NEO-FFI; Costa and McCrae) developed by Zawadzki, Strelau, Szczepaniak, and Śliwińska [4,5,21]. The NEO-FFI comprises 60 items, with 12 items assessing each of the five personality domains: neuroticism, extraversion, openness to experience, agreeableness, and conscientiousness. The Polish adaptation has demonstrated satisfactory reliability and factorial validity and provides age- and sex-specific sten norms [21].

Illness acceptance was assessed using the Polish adaptation of the eight-item Acceptance of Illness Scale (AIS), originally developed by Felton, Revenson, and Hinrichsen and adapted to Polish conditions by Juczyński [20,22]. The total AIS score ranges from 8 to 40, with higher scores indicating greater illness acceptance. Previous studies have supported the reliability and applicability of the Polish AIS among patients with chronic diseases, including cancer [22,23]. According to oncology-specific Polish norms, scores of 8–18 indicate low illness acceptance, scores of 19–36 indicate average illness acceptance, and scores of 37–40 indicate high illness acceptance [23]. Perceived social support was assessed using a single study-specific item with the response options “feels supported”, “difficult to say”, and “does not feel supported”; no participant selected the last option. Self-rated treatment outcome was assessed using a single study-specific item with five response categories ranging from “very poor” to “very good”. Because only two participants selected “poor” and none selected “very poor”, the “poor” responses were combined with the “average” category for group comparisons.

### 2.3. Data Analysis

Analyses were conducted in R (version 4.2.2) via RStudio (version 2022.12.0), with the significance threshold set at α = 0.05. Shapiro–Wilk tests indicated that all five NEO-FFI domain scores departed significantly from normality (all *p* < 0.05), whereas normality was not rejected for the AIS total score (W = 0.978, *p* = 0.053). Non-parametric methods were retained because several study variables were non-normally distributed and the study-specific measures were ordinal. All reported associations were unadjusted bivariate associations. Effect sizes—eta-squared for Kruskal–Wallis, epsilon-squared for Mann–Whitney—accompany each significant test. Given the exploratory character of the study, no correction for multiple testing was applied.

## 3. Results

### 3.1. Characteristics of Study Group

**Figure 1:**
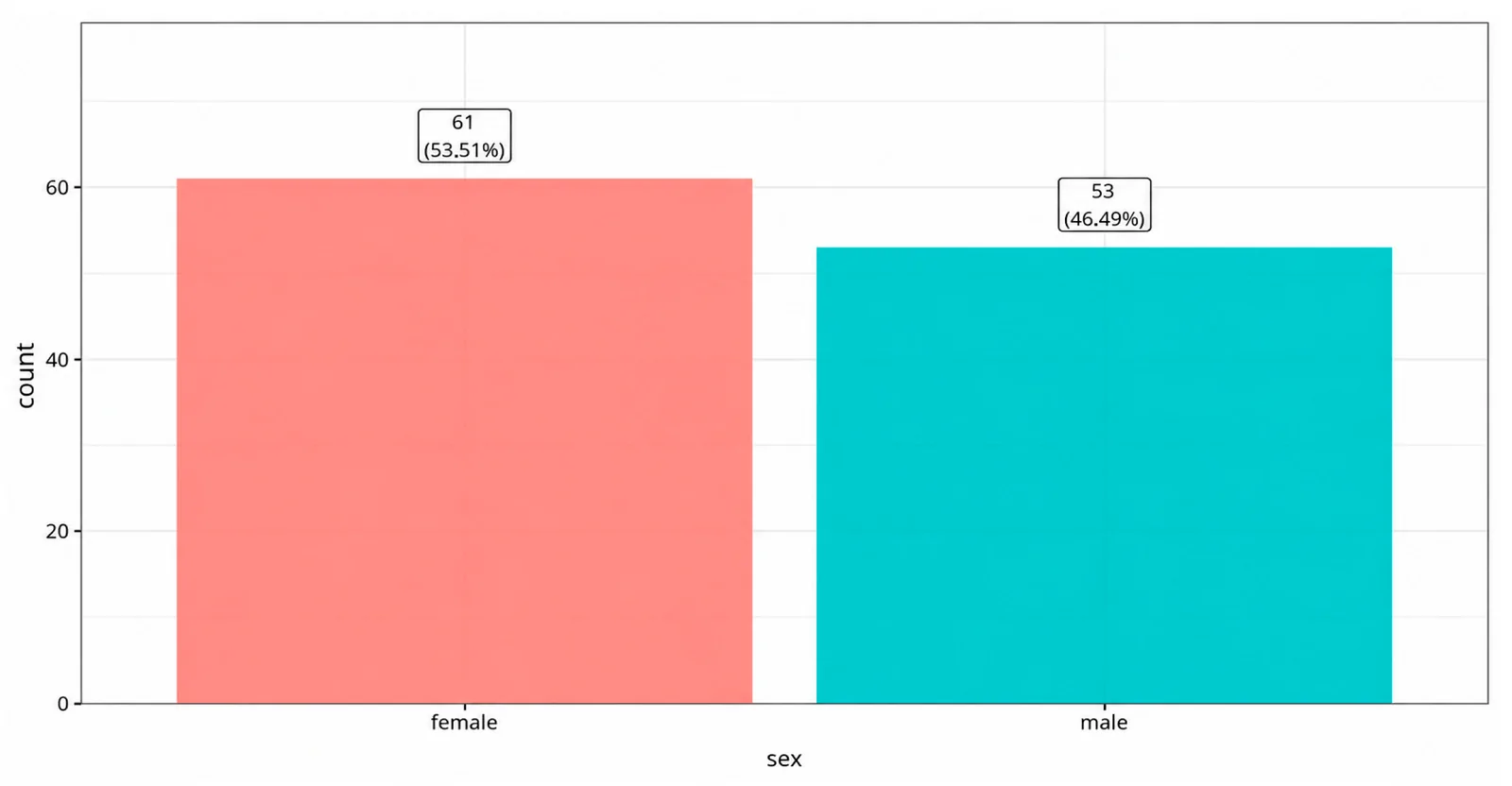
The sex distribution of the 114 study participants.

**Figure 2:**
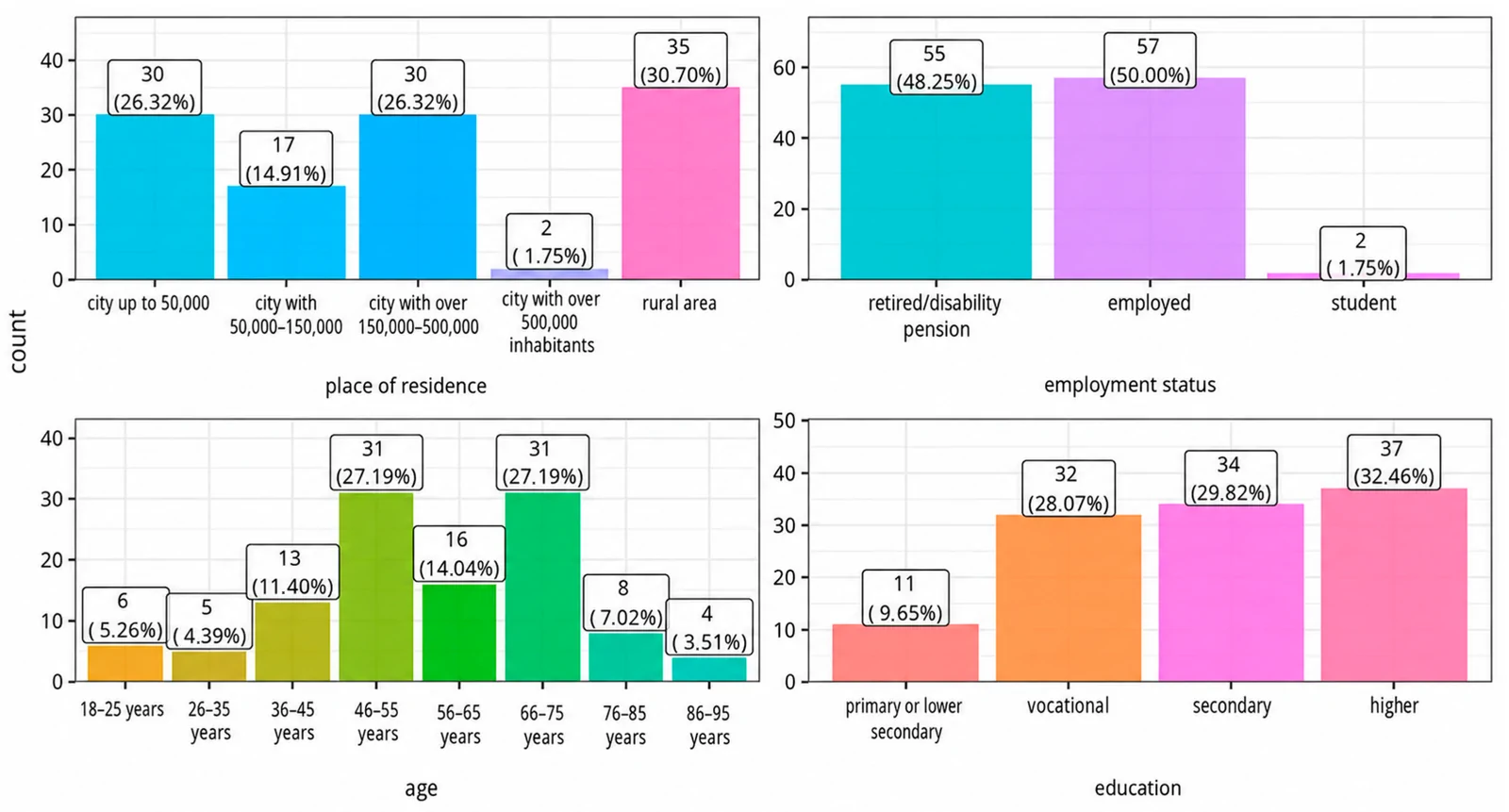
Age, place of residence, education level, and occupational situation among study participants.

Women were slightly more numerous than men in the sample of 114 patients (Figure 1). Two age bands—46–55 and 66–75—each accounted for roughly a quarter of participants; about one in seven was aged 56–65, with smaller groups spread across the remaining bands and the fewest patients aged 86–95. Residentially, fewer than a third lived in a rural area, about a quarter each in a small town or a mid-sized city (150,000–500,000 residents), one in seven in a city of 50,000–150,000, and the smallest group in a city exceeding 500,000. Half of the sample was professionally active, with retirees, disability pensioners, and a small group of students making up the remainder; educational attainment skewed toward higher and secondary education, with vocational and primary/lower-secondary education less common (Figure 2).

Table 1 lists the tumor sites reported by participants. Breast cancer was the single most common diagnosis, followed by prostate, colorectal, and lung cancer; kidney, male breast, and cervical cancer each accounted for a smaller but comparable share; liver, ovarian, and testicular cancer were less common still; and a long tail of diagnoses—including esophageal, skin, adrenal, bladder, thyroid, gastric, small-intestinal, nasal septum, and several rarer sites—each affected only a handful of patients.

Disease duration averaged 4.25 years (SD = 3.28; median = 3.00 years), ranging from under a year in the most recently diagnosed patient to 16 years in the longest-standing case (Table 2).

Self-assessed treatment outcome skewed favorable (Table 3): no patient rated their outcome as very poor, and only two rated it poor; the remainder split fairly evenly between average, good, and very good, with good the single most common rating.

**Figure 3:**
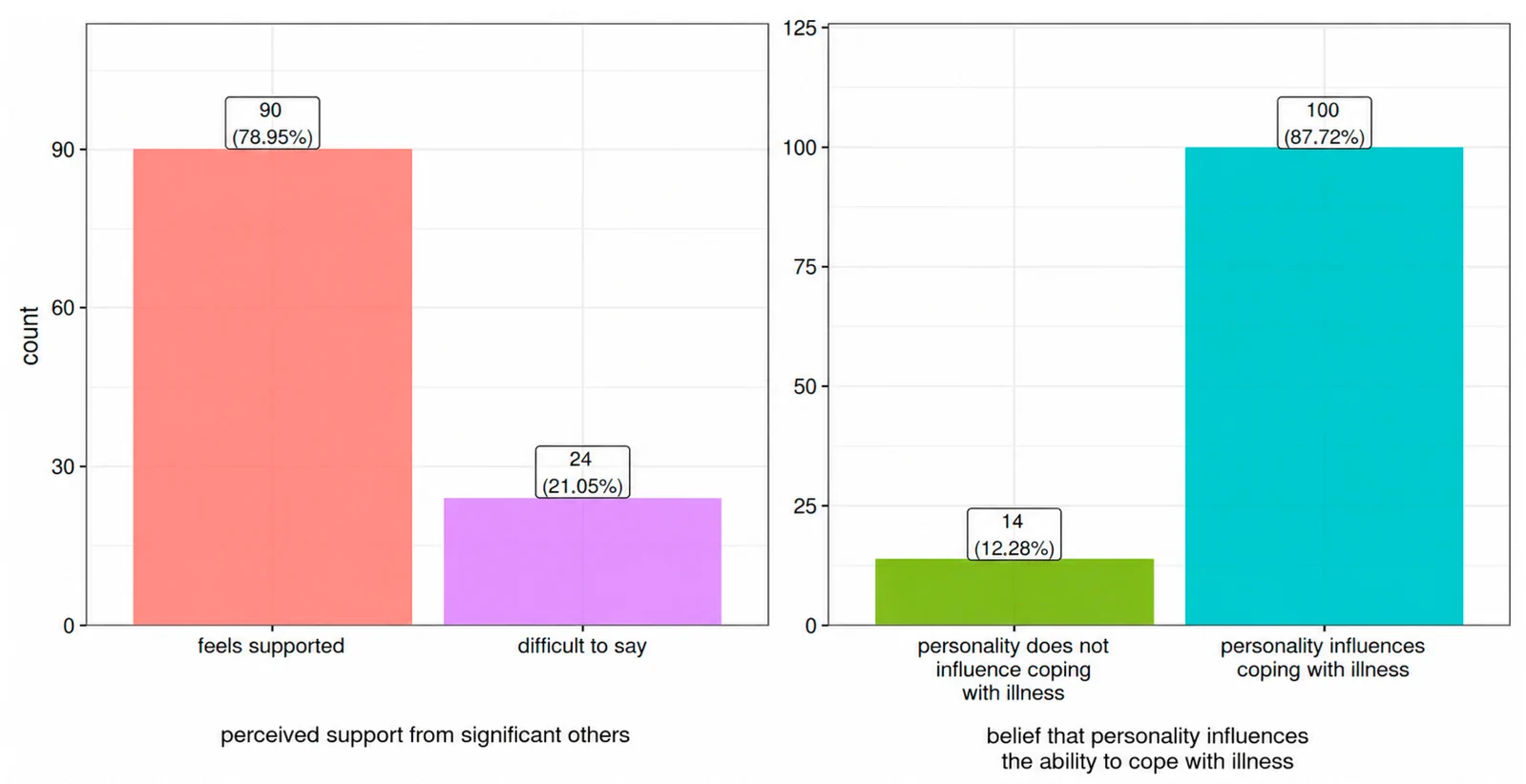
Perceived support from close relatives and beliefs about the role of personality in coping with cancer (n = 114).

Turning to perceived support and beliefs about personality (Figure 3), the large majority of patients reported feeling supported by close relatives, with about one in five finding this difficult to judge and no patient reporting an absence of support altogether. A similarly large majority believed that personality shapes how well someone copes with cancer, against roughly one in ten who saw no such influence.

### 3.2. Personality Profile

Patients scored, on average, within the moderate range on all five NEO-FFI domains, with agreeableness highest, followed by conscientiousness, neuroticism, and extraversion, and openness to experience lowest (Table 4).

### 3.3. Personality, Disease Duration, Social Support, and Treatment Outcome

**Figure 4:**
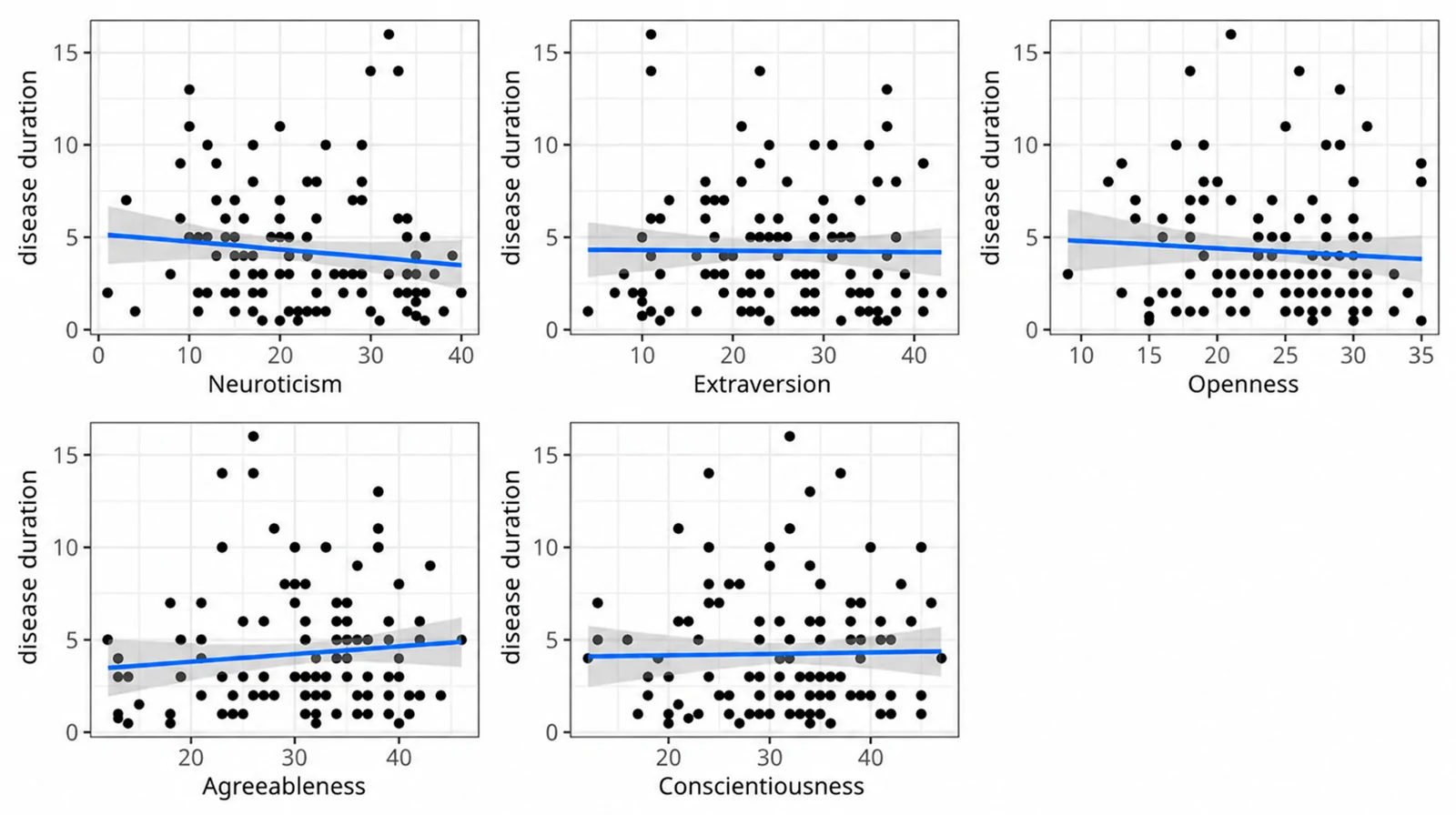
Association between disease duration and NEO-FFI personality domains.

Disease duration correlated weakly and negatively with neuroticism (ρ = −0.191, *p* = 0.041); none of the other four domains showed a significant relationship with duration (extraversion ρ = −0.019, *p* = 0.840; openness ρ = −0.077, *p* = 0.416; agreeableness ρ = 0.126, *p* = 0.182; conscientiousness ρ = 0.042, *p* = 0.656) (Figure 4).

Perceived support from close relatives, by contrast, distinguished patients on every personality domain (Table 5): those who felt supported scored significantly lower on neuroticism and significantly higher on extraversion, openness, agreeableness, and conscientiousness than those who found it difficult to judge whether they were supported (Mann–Whitney U tests, all *p* < 0.05), with effect sizes ranging from moderate for neuroticism and extraversion to large for agreeableness and conscientiousness.

Self-assessed treatment outcome likewise differentiated four of the five domains (Table 6; the two patients rating their outcome “poor” were combined with the “average” category because of the small cell size). Neuroticism showed the strongest gradient—lowest among patients rating their outcome very good, highest among those rating it average (Kruskal–Wallis H = 18.134, df = 2, *p* < 0.001, η^2^ = 0.145)—mirrored by similarly strong, opposite-direction gradients for agreeableness (H = 14.669, *p* = 0.001, η^2^ = 0.114) and conscientiousness (H = 13.126, *p* = 0.001, η^2^ = 0.100). Extraversion showed a weaker but still significant gradient (H = 6.711, *p* = 0.035, η^2^ = 0.042); openness did not differ significantly across treatment-outcome categories (H = 3.121, *p* = 0.210).

### 3.4. Level of Illness Acceptance

The mean AIS score was 26.55 (SD = 7.34; range 8–40) (Table 7). Two-thirds of patients (66.67%, 95% confidence interval (CI): 58.01–75.32%) fell in the average acceptance band, 14.04% (95% CI: 7.66–20.41%) in the high band, and 19.30% (95% CI: 12.05–26.54%) in the low band.

### 3.5. Illness Acceptance, Disease Duration, Social Support, and Treatment Outcome

**Figure 5:**
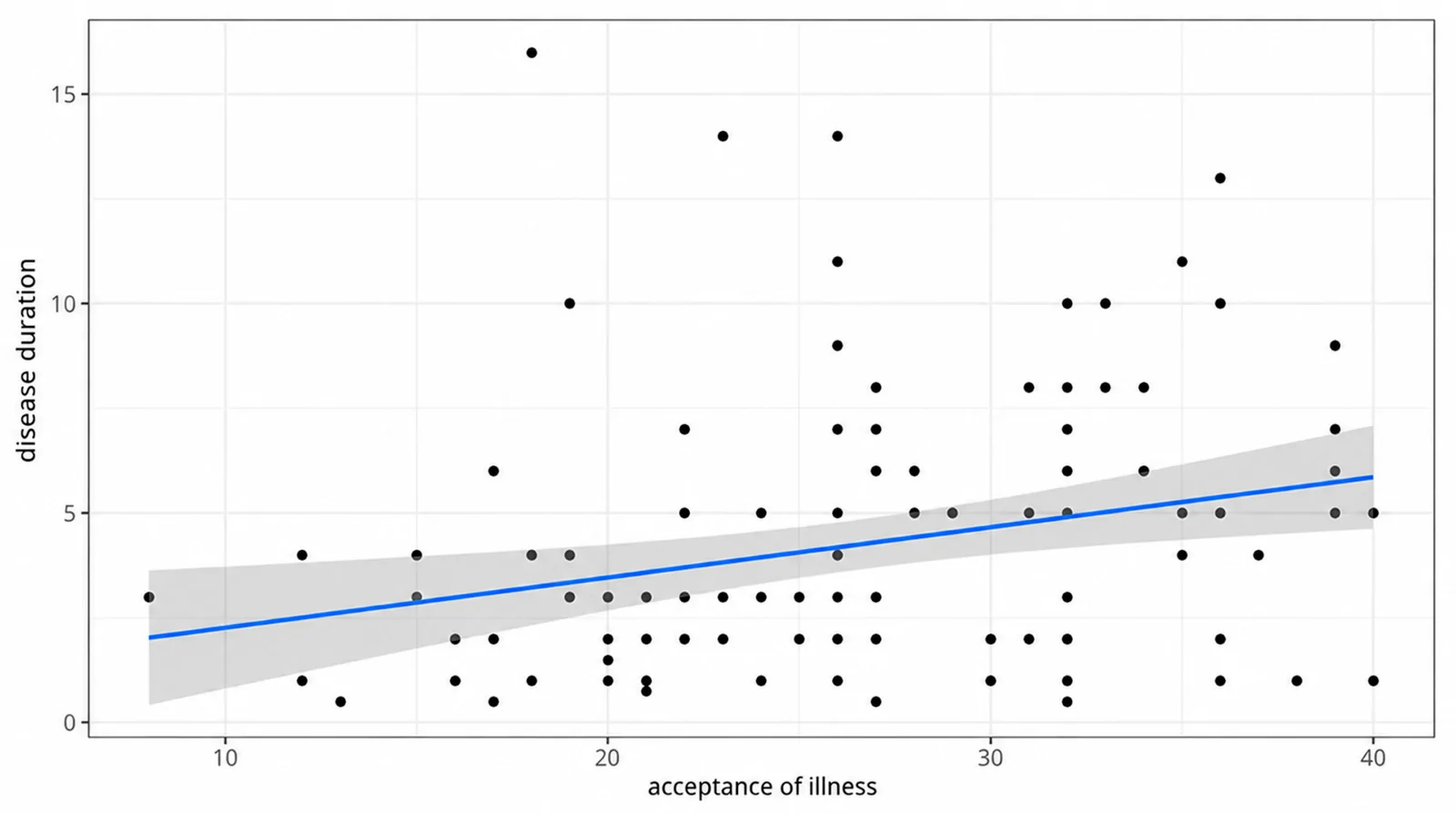
Association between disease duration and illness acceptance (AIS).

Illness acceptance correlated moderately and positively with disease duration (ρ = 0.332, *p* < 0.001): patients further out from diagnosis reported higher acceptance (Figure 5).

Patients who felt supported by close relatives scored significantly, if modestly, higher on the AIS than those who found this difficult to judge (Mann–Whitney U = 1371.5, *p* = 0.043, ε^2^ = 0.036) (Table 8).

Self-assessed treatment outcome showed a strong, graded relationship with acceptance (Kruskal–Wallis H = 17.834, degrees of freedom (df) = 2, *p* < 0.001, η^2^ = 0.143): acceptance was highest among patients rating their outcome very good, intermediate among those rating it good, and lowest among those rating it average; post hoc tests confirmed the “very good” group differed significantly from both the “good” (*p* = 0.007) and “average” (*p* < 0.001) groups (Table 9).

### 3.6. Personality and Illness Acceptance

**Figure 6:**
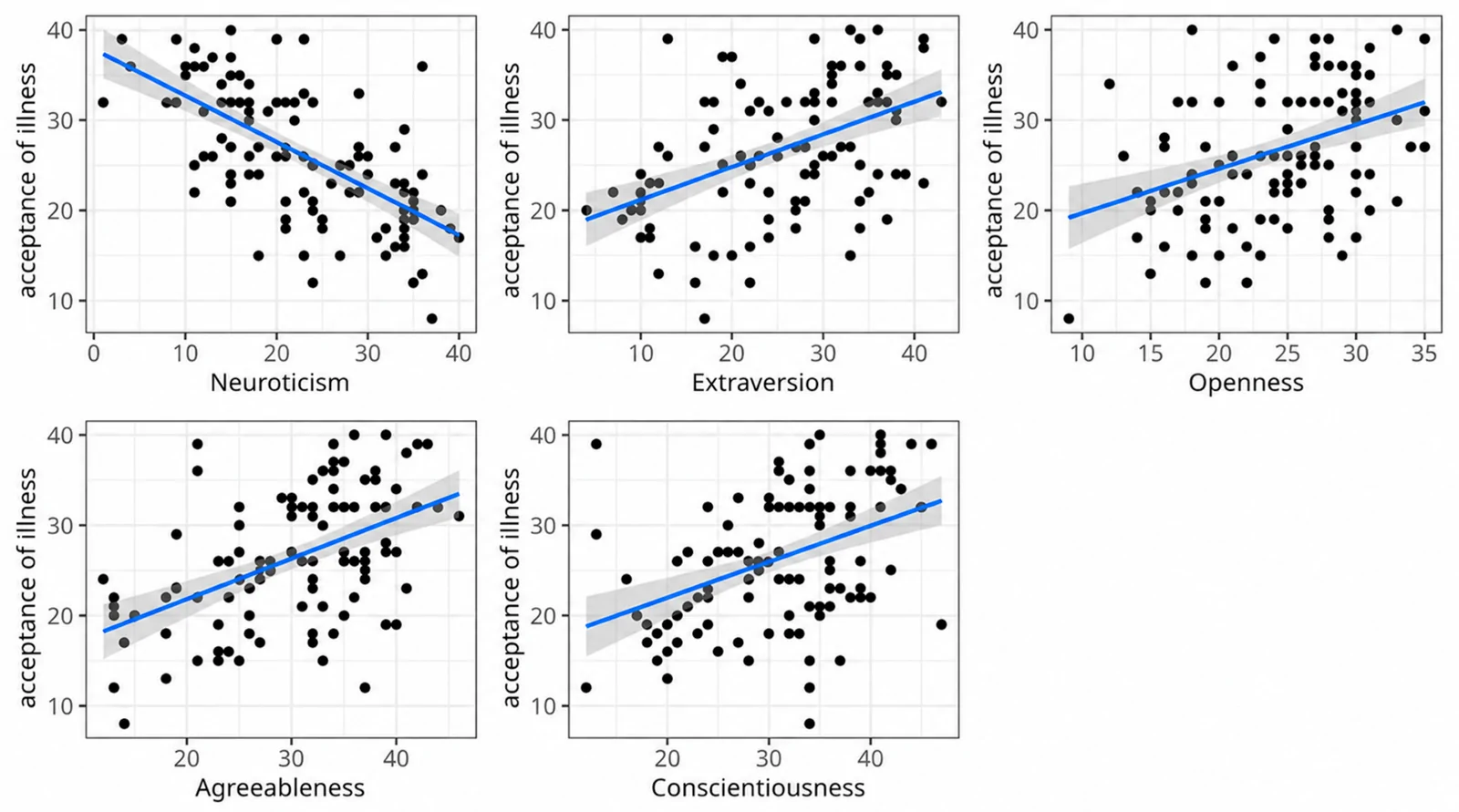
Association between NEO-FFI personality domains and illness acceptance (AIS).

Every one of the five NEO-FFI domains correlated significantly with illness acceptance (Table 10, Figure 6). The relationship was strongest, and negative, for neuroticism (ρ = −0.632, *p* < 0.001); all other domains correlated moderately and positively—extraversion (ρ = 0.459, *p* < 0.001), agreeableness (ρ = 0.452, *p* < 0.001), conscientiousness (ρ = 0.421, *p* < 0.001), and openness to experience (ρ = 0.367, *p* < 0.001).

## 4. Discussion

Every one of the five NEO-FFI domains was significantly associated with illness acceptance in this sample, but not equally so: neuroticism dwarfed the other four in effect size, and its direction—higher neuroticism, lower acceptance—was unambiguous. That pattern places the present findings squarely within a now-substantial body of evidence identifying neuroticism as the personality domain most reliably implicated in poor psychological adjustment to cancer. In the largest study of its kind, Morgan and colleagues found that a “Distressed” personality profile—defined chiefly by high neuroticism—carried significantly more depression, anxiety, and symptom burden than profiles characterized by lower neuroticism [11], and follow-up work on the same cohort tied that profile to a narrower, more disengaged coping repertoire [12] and to worse trajectories of stress and resilience over the course of chemotherapy [13]. Smaller studies point the same direction: neuroticism has been linked to greater demoralization in end-of-life cancer care [14] and to elevated distress in breast cancer patients scoring high on Type-D personality, a construct that substantially overlaps with high neuroticism and low extraversion [15].

Extraversion, agreeableness, and conscientiousness all moved in the opposite direction from neuroticism, and all three showed their strongest, most consistent associations not with time since diagnosis but with perceived social support and self-assessed treatment outcome—domains where interpersonal and self-regulatory skills plausibly matter most directly. Patients who scored higher on these three traits were both more likely to feel supported by close relatives and more likely to rate their treatment as successful, a pattern consistent with a straightforward interpretive account: extraverted, agreeable patients may find it easier to mobilize and sustain supportive relationships during treatment, while conscientious patients may adhere more consistently to medical recommendations and therefore experience—or at least perceive—better outcomes. Conscientiousness’s broader reputation as the personality domain most tightly coupled to health-protective behavior [10] fits comfortably with this account, as does its documented role, together with the other Five-Factor domains, in shaping vulnerability to psychiatric symptoms more generally [8,9].

Openness to experience was the one domain whose association with acceptance did not fit a simple “Resilient-profile-good, Distressed-profile-bad” narrative: it correlated positively with acceptance yet showed no significant relationship with treatment-outcome ratings, and was itself implicated, alongside neuroticism, as a risk factor for breast cancer diagnosis in a recent case–control study [16]. This dissociation is a reminder that the Five-Factor domains do not always move in lockstep across different cancer-related outcomes, and that openness in particular may carry different—sometimes opposing—implications depending on whether the outcome in question is disease risk, treatment response, or, as here, subjective acceptance.

Disease duration and perceived treatment success were, alongside personality, the two strongest correlates of acceptance identified in this study, and both make intuitive sense through the lens of the common-sense model of illness representation, which holds that patients build cognitive and emotional models of their condition—its likely course, controllability, and consequences—that guide subsequent coping and adjustment [19]. Longer-diagnosed patients have had more time to incorporate cancer into a stable self-concept, and patients who believe their treatment is working have correspondingly less reason to resist the diagnosis psychologically. Perceived support from close relatives was also associated with higher illness acceptance in the unadjusted group comparison, consistent with the broader literature describing social support as an important psychosocial resource in oncology.

The mean AIS score observed here, and the moderate-acceptance profile it implies for most patients, is broadly consistent with earlier Polish oncology samples using the same instrument: a study of 229 randomly selected patients at a specialist oncology hospital reported a comparable mean AIS score and similarly identified age, marital status, and occupational activity as correlates of acceptance [24], suggesting that the moderate-acceptance pattern reported here is not an artifact of this particular clinic or patient mix.

Neuroticism’s outsized role in this dataset also echoes findings well outside oncology. A study of 246 men with coronary heart disease, using the same NEO-FFI instrument, identified neuroticism as the single most important predictor of poor psychosocial adjustment, with the best-adjusted patients combining low neuroticism with high extraversion [25]—a personality signature strikingly similar to the one associated with high illness acceptance in the present cancer sample. Emotional stability, in other words, appears to function as a broadly transdiagnostic resource for adjusting to serious chronic illness, cancer included.

Two much older strands of psycho-oncology theory are worth revisiting in light of these results. Temoshok’s Type C construct—emotionally suppressive, excessively compliant, self-effacing—was proposed specifically as a cancer-relevant personality style [2], and the Mental Adjustment to Cancer framework built an entire assessment tradition around cognitive-behavioral response styles such as fighting spirit and helplessness [3]. Neither maps perfectly onto the Five-Factor Model, but both anticipate, in earlier and less standardized language, the same general pattern reported here: a patient’s characteristic way of engaging emotionally with the world was associated with how well they had come to terms with a cancer diagnosis.

Taken together, these findings argue for treating personality as more than background noise in oncology care. A patient presenting with high neuroticism and low extraversion is, on this evidence, measurably less likely to have accepted their diagnosis months or years after receiving it—a vulnerability that could plausibly be identified early and addressed with targeted psychological support rather than left to resolve, or not, on its own.

Although personality traits are relatively stable, illness acceptance should not be understood as being determined by personality alone. Psychological adjustment to cancer develops within a broader biopsychosocial context that includes treatment-related symptoms, coping strategies, social resources, and potentially modifiable health behaviors such as physical activity, diet, sleep, and smoking. Population-based cross-sectional research has shown that better adherence to Life’s Essential 8 health behaviors is associated with lower odds of depression [26]. Although that study involved a different population and outcome, it illustrates the potential relevance of modifiable behaviors to mental health. Accordingly, personality assessment should be regarded as one component of a broader psychosocial evaluation and as a means of guiding supportive and behavioral interventions rather than as a fixed indicator of a patient’s capacity to adjust.

### Limitations

Several features of this study limit how far its findings can be generalized. Its cross-sectional design cannot establish whether personality shapes acceptance; whether a long, difficult illness course reshapes personality; or both simultaneously. The sample was drawn from a single diagnostic-imaging department and spans a wide range of tumor sites and, presumably, disease stages that were not captured or analyzed as potential effect modifiers; future work should stratify by cancer type and stage. Clinical variables such as treatment modality, comorbidity burden, and objective disease staging were not collected, limiting the ability to rule out confounding. The principal strength of the study is methodological: two well-validated instruments applied to a diagnostically heterogeneous, reasonably large sample of oncology outpatients, generating a multidimensional picture of how personality, disease course, and social context jointly relate to illness acceptance within a single population. Although the Polish versions of the NEO-FFI and AIS have demonstrated satisfactory reliability in previous validation studies, internal consistency was not re-estimated in the present sample. In addition, perceived social support and self-rated treatment outcome were each assessed using a single study-specific item rather than a validated multidimensional instrument. These measures may be affected by measurement error, current mood, and individual interpretation, while self-rated treatment outcome should not be regarded as an objective clinical measure of treatment effectiveness. The analyses were unadjusted and bivariate, and no correction for multiple testing was applied. Therefore, residual confounding cannot be excluded, and findings with *p* values close to 0.05 should be interpreted as exploratory and with particular caution.

## 5. Conclusions

1. Illness acceptance was moderate on average; 19.30% of participants were classified as having low acceptance, 66.67% as average acceptance, and 14.04% as high acceptance.
2. All five Five-Factor personality domains were significantly associated with illness acceptance, with neuroticism showing by far the strongest—and negative—association.
3. Extraversion, agreeableness, conscientiousness, and openness to experience were all positively associated with acceptance, though openness stood apart in showing no relationship with self-assessed treatment outcome.
4. Longer disease duration and a more favorable self-rated treatment outcome were each associated with higher illness acceptance in separate unadjusted analyses.
5. Perceived support from close relatives was associated with both higher acceptance and a markedly more favorable personality profile across all five domains, underscoring social context as a variable closely intertwined with both personality expression and psychological adjustment.
6. These findings support incorporating brief psychosocial assessment into routine oncology care, including consideration of personality-related vulnerability alongside potentially modifiable behavioral and social factors. Particular attention may be warranted for patients presenting with high neuroticism and low extraversion, while avoiding the interpretation that psychological adjustment is determined by personality alone.

## Figures and Tables

**Table 1:** Tumor site among study participants (n = 114).

Tumor Site	n	%
Female Breast	16	14.04
Prostate Gland	10	8.77
Colon (Large Intestine)	9	7.89
Lung	9	7.89
Kidney	8	7.02
Male Breast	8	7.02
Cervix	8	7.02
Liver	6	5.26
Ovary	5	4.39
Testis	5	4.39
Esophagus	4	3.51
Skin	4	3.51
Adrenal Gland	3	2.63
Urinary Bladder	3	2.63
Thyroid	3	2.63
Stomach	3	2.63
Small Intestine	2	1.75
Nasal Septum	2	1.75
Leukemia	1	0.88
Head	1	0.88
Tongue	1	0.88
Larynx	1	0.88
Pancreas	1	0.88
Gallbladder	1	0.88

n—number of observations; %—percentage.

**Table 2:** Disease duration among study participants (n = 114).

Variable	M	SD	Me	Min	Max	Skewness	Kurtosis	S–W (W)	*p*
Disease duration (years)	4.25	3.28	3.00	0.5	16	1.248	1.365	0.878	<0.001

M—mean; Me—median; SD—standard deviation; S–W—Shapiro–Wilk test.

**Table 3:** Self-assessed cancer treatment outcome (n = 114).

Very Poor	Poor	Average	Good	Very Good	Me
0 (0.00%)	2 (1.75%)	27 (23.68%)	49 (42.98%)	36 (31.58%)	4

Me—median.

**Table 4:** NEO-Five-Factor Inventory (NEO-FFI) raw and sten score descriptives (n = 114).

Personality Domain	M (Raw)	SD	Me	Min	Max	M (Sten)	SD (Sten)
Neuroticism	22.06	8.97	21.0	1	40	5.39	2.37
Extraversion	25.01	9.40	25.5	4	43	5.18	2.74
Openness to experience	24.04	5.84	25.0	9	35	4.95	2.19
Agreeableness	30.57	7.90	32.0	12	46	5.77	2.84
Conscientiousness	31.53	7.84	32.0	12	47	5.64	2.47

M—mean; Me—median; SD—standard deviation. All five raw-score distributions departed significantly from normality (Shapiro–Wilk, *p* < 0.05).

**Table 5:** NEO-FFI domains by perceived support from close relatives (Mann–Whitney U test).

Personality Domain	Support Group	n	M	SD	Me	Mr	U	*p*	ε^2^
Neuroticism	Feels supported	90	20.86	8.51	20.0	53.32	703.5	0.009	0.061
	Difficult to say	24	26.58	9.39	29.5	73.19			
Extraversion	Feels supported	90	26.20	9.53	28.0	61.85	1471.5	0.007	0.066
	Difficult to say	24	20.54	7.54	21.5	41.19			
Openness to experience	Feels supported	90	24.92	5.47	26.0	62.36	1517.5	0.002	0.082
	Difficult to say	24	20.75	6.11	20.0	39.27			
Agreeableness	Feels supported	90	32.40	6.46	33.0	63.91	1656.5	<0.001	0.142
	Difficult to say	24	23.71	9.11	23.0	33.48			
Conscientiousness	Feels supported	90	33.10	7.50	34.0	64.48	1708.0	<0.001	0.169
	Difficult to say	24	25.62	6.14	24.5	31.33			

n—number of observations; M—mean; SD—standard deviation; Me—median; Mr—mean rank; U—Mann–Whitney U statistic; ε^2^—epsilon-squared effect size.

**Table 6:** NEO-FFI domains by self-assessed treatment outcome (Kruskal–Wallis H test).

Personality Domain	Treatment Outcome	n	M	SD	Me	Mr	H	df	*p*	η^2^
Neuroticism	Very good	36	17.94	7.50	15.5	41.29	18.134	2	<0.001	0.145
	Good	49	21.96	7.71	21.0	58.27				
	Average	29	27.34	10.11	31.0	76.33				
Extraversion	Very good	36	28.06	8.09	29.0	68.21	6.711	2	0.035	0.042
	Good	49	24.71	9.13	25.0	55.68				
	Average	29	21.72	10.44	22.0	47.28				
Openness	Very good	36	24.42	5.95	25.0	59.07	3.121	2	0.210	0.010
	Good	49	24.71	5.70	26.0	61.76				
	Average	29	22.45	5.83	23.0	48.36				
Agreeableness	Very good	36	34.42	5.38	34.0	73.56	14.669	2	0.001	0.114
	Good	49	30.06	7.73	31.0	54.38				
	Average	29	26.66	8.84	30.0	42.84				
Conscientiousness	Very good	36	35.00	5.94	35.0	71.69	13.126	2	0.001	0.100
	Good	49	31.06	7.83	32.0	56.26				
	Average	29	28.00	8.35	27.0	41.98				

n—number of observations; M—mean; SD—standard deviation; Me—median; Mr—mean rank; H—Kruskal–Wallis H statistic; df—degrees of freedom; η^2^—eta-squared effect size.

**Table 7:** Acceptance of Illness Scale (AIS) scores (n = 114); distribution of illness acceptance levels (n = 114).

Variable	M	SD	Me	Min	Max	Skewness	Kurtosis	S-W (W)	*p*
AIS total score	26.55	7.34	26	8	40	−0.094	−0.794	0.978	0.053
**Level of Illness Acceptance**		**n**		**%**		**95% CI Lower**		**95% CI Upper**	
Low		22		19.30		12.05		26.54	
Average		76		66.67		58.01		75.32	
High		16		14.04		7.66		20.41	

M—mean; Me—median; SD—standard deviation; S–W—Shapiro–Wilk test. CI—confidence interval. AIS categories were defined according to oncology-specific Polish norms: low, 8–18 points; average, 19–36 points; and high, 37–40 points [23].

**Table 8:** Illness acceptance (AIS) by perceived support from close relatives (Mann–Whitney U test).

Support Group	n	M	SD	Me	Mr	U	*p*	ε^2^
Feels supported	90	27.38	7.03	26.5	60.74	1371.5	0.043	0.036
Difficult to say	24	23.46	7.84	22.0	45.35			

n—number of observations; M—mean; SD—standard deviation; Me—median; Mr—mean rank; U—Mann–Whitney U statistic; ε^2^—epsilon-squared effect size.

**Table 9:** Illness acceptance (AIS) by self-assessed treatment outcome (Kruskal–Wallis H test).

Treatment Outcome	n	M	SD	Me	Mr
Very good	36	30.47	7.16	32	74.99
Good	49	25.86	5.96	26	54.44
Average	29	22.86	7.61	21	40.97

H = 17.834; df = 2; *p* < 0.001; η^2^ = 0.143.

**Table 10:** Correlations between NEO-FFI personality domains and illness acceptance (AIS) (Spearman’s rho).

Personality Domain	ρ	*p*
Neuroticism	−0.632	<0.001 *
Extraversion	0.459	<0.001 *
Openness to experience	0.367	<0.001 *
Agreeableness	0.452	<0.001 *
Conscientiousness	0.421	<0.001 *

* *p* < 0.05. ρ—Spearman’s rank correlation coefficient.

## Data Availability

The data presented in this study are available on request from the corresponding author.
